# Supplementary material for: Architecture of the baculovirus nucleocapsid revealed by cryo-EM
Source: Nat Commun. 2023 Nov 18;14:7481. doi: 10.1038/s41467-023-43284-1 (PMC10657434; doi:10.1038/s41467-023-43284-1)
Supplement: Supplementary file 1 — Supplementary Information [file 41467_2023_43284_MOESM1_ESM.pdf]

Supplementary information

Architecture of the baculovirus nucleocapsid revealed by cryo-EM

Xudong Jia[1#], Yuanzhu Gao[1,2#], Yuxuan Huang[1], Linjun Sun[1], Siduo Li[1], Hongmei Li[1], Xueqing Zhang[1], Yinyin Li[1], Jian He[1], Wenbi Wu[1], Harikanth Venkannagari[3], Kai Yang[1], Matthew L. Baker[3*], Qinfen Zhang[1*]

1 State key lab for biocontrol, School of Life Sciences, Sun Yat-sen University, Guangzhou, China, 510275

2 Cryo-EM Facility Center, Southern University of Science and Technology, Shenzhen, China

3 Department of Biochemistry and Molecular Biology, McGovern Medical School at the University of Texas Health Science Center, Houston, TX 77030

# These authors contributed equally to this work.

* To whom correspondence may be addressed to: M.L.B (Matthew.L.Baker@uth.tmc.edu); and Q.Z (Lsszqf@mail.sysu.edu.cn)

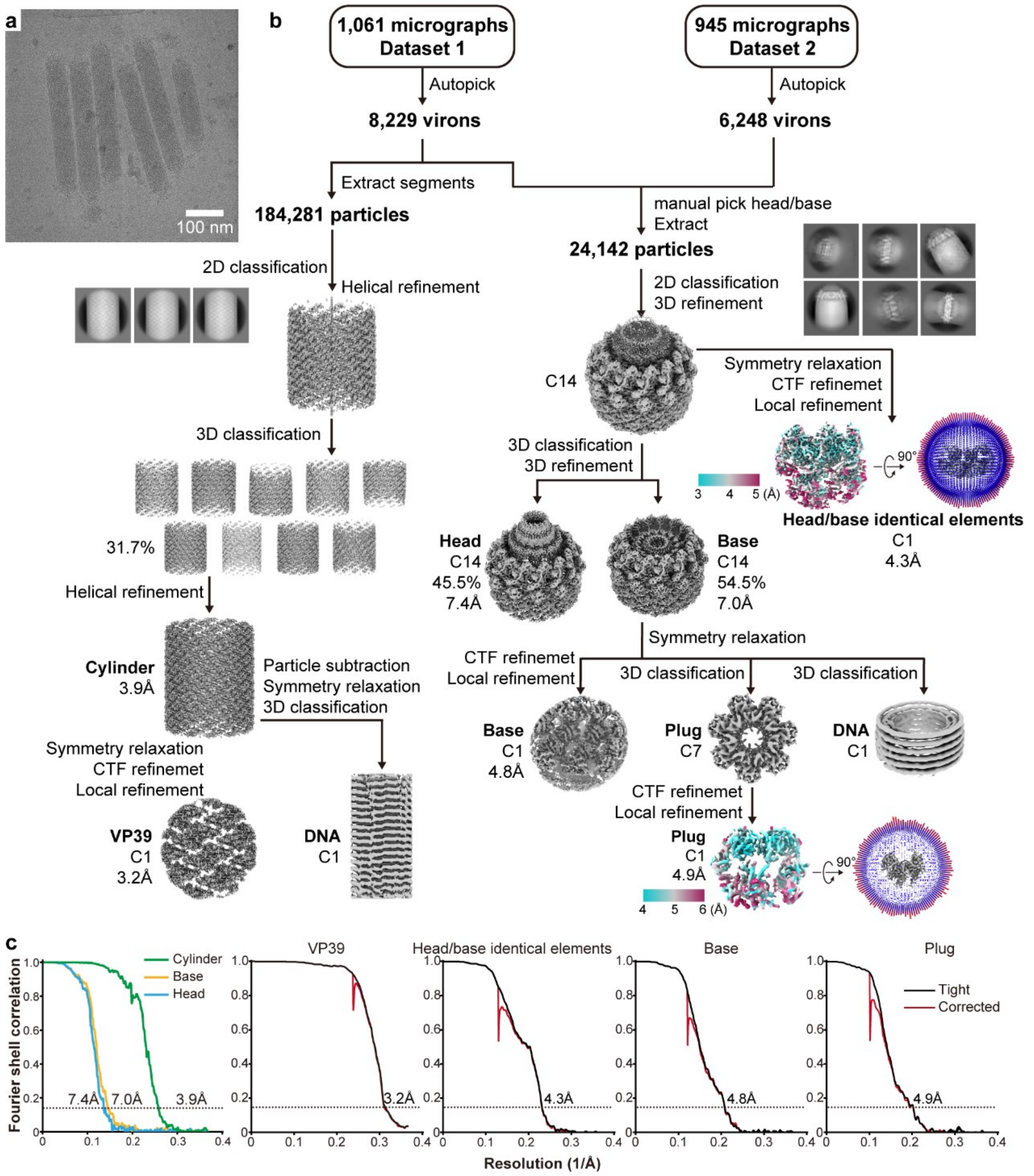

Supplementary Figure 1. Reconstruction strategy in AcMNPV.

a, Typical AcMNPV ODV nucleocapsids particles selected from more than 2000 cryo-EM micrographs. **b,** Reconstruction strategy flowchart with symmetry, 2D classes, particle numbers, angle distribution of the final selected particle, local resolution (cut-off 0.143) colored map are indicated. **c,** Resolution assessments of the final maps using the gold standard FSC criteria at 0.143.

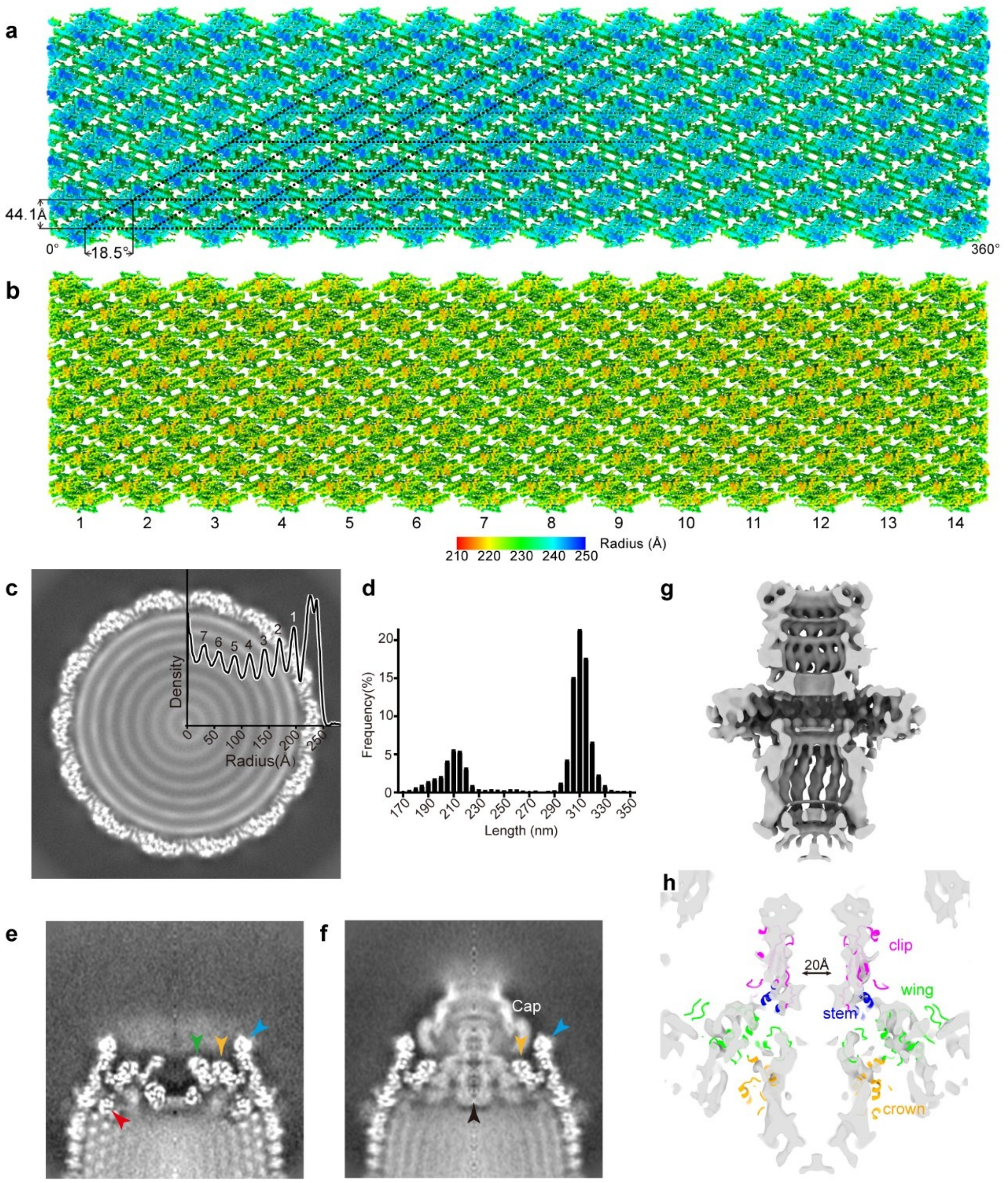

Supplementary Figure 2. Structural features of the AcMNPV nucleocapsid.

a, Planar display of unwrapped helical cylinder viewed from outside, after masking away the genome density. The helical cylinder has 14-fold symmetry with a rotation angle of 18.5° and rise of 44.1 Å per asymmetric unit. **b,** Planar display of unwrapped helical cylinder viewed from inside. A scale bar is shown for both panel **(a)** and **(b)**. **c,** One cross-section of the nucleocapsid helical cylinder demonstrates at least 7 layers of dsDNA enclosed by the helical cylinder, whose radius is ~ 250 Å. **d,** The length distribution of AcMNPV helical cylinder (capsid's inner cavity) is shown (n=2,503). Source data are provided as a Source Data file. There are two peaks: 210nm and 310nm. **e,** A central longitudinal slice of the base. The red arrow indicates the additional density compared to the apical portion of the nucleocpasid. The blue arrow indicates the outer shell. The orange arrows indicate the inner layer, and green arrow corresponds to the plug. **f,** The central longitudinal slice of head and cap. The cap sits at the tip of the capsid. The blue arrow and orange arrow indicate the outer shell and inner layer, respectively. In the central portion of the head, there is a portal-like structure (black arrow). **g,** The cutaway side view of the portal-like structure of AcMNPV. **h,** A slice of the portal-like structure shows the density superimposed with the portal structure from the T4 phage (PDB: 3JA7)[1].

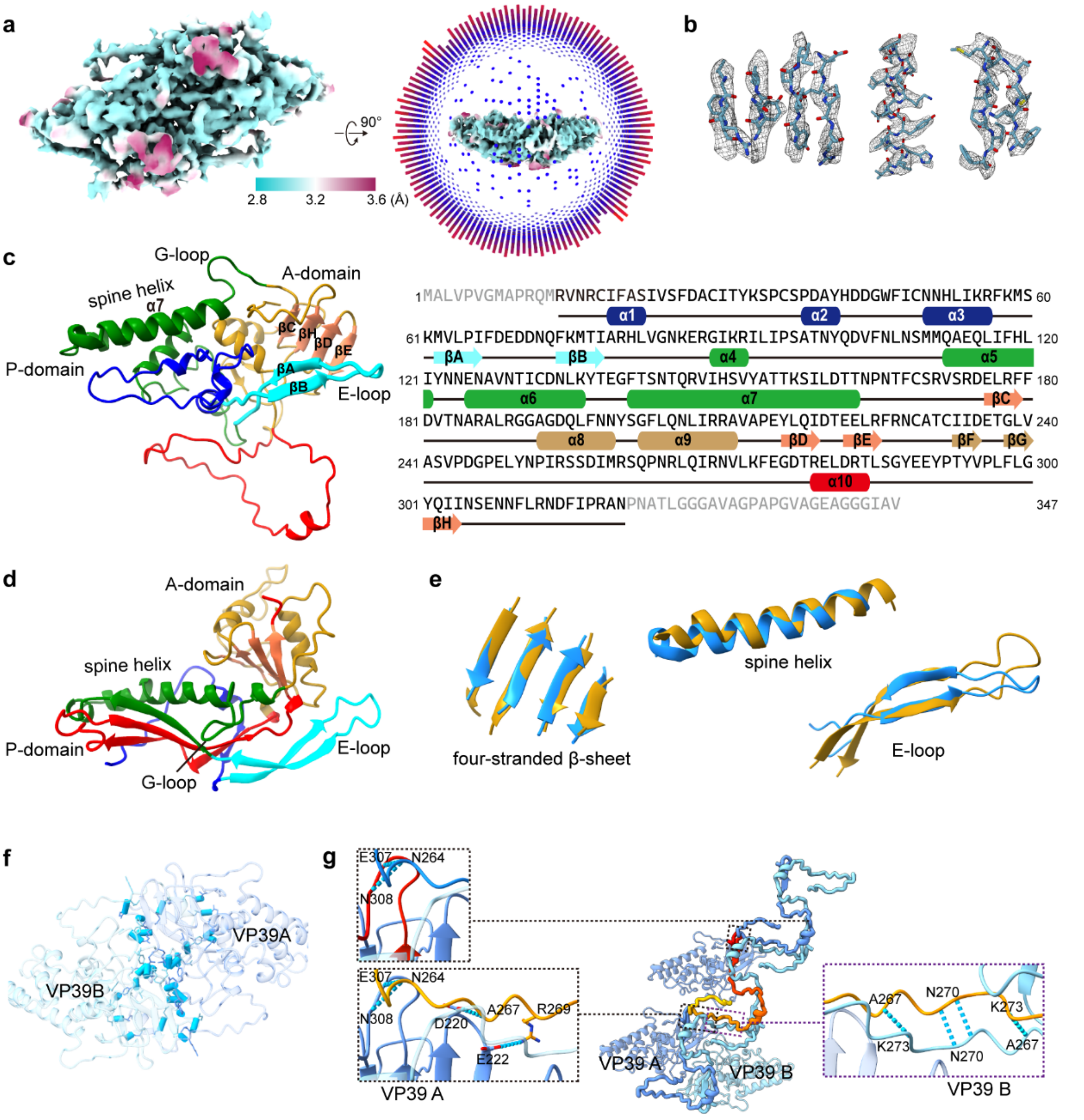

Supplementary Figure 3. Structural features of VP39.

a, VP39 dimer viewed from outside (left) showing the local resolution (cut-off 0.143), as well as the angle distributions of selected particles (right) are indicated. **b,** Representative segments of the atomic model (ribbon) superimposed with corresponding densities of VP39 are shown. **c,** The model of VP39, as viewed from inside, is shown. The structure has elements in common with the HK97-fold. The N-terminal region is colored in blue, the E-loop is colored in cyan, the long “spine helix” in the P-domain is colored green. The A-domain is colored orange, and the C-terminal region is colored in red. In the right panel, the sequence and secondary structural elements are indicated. The color schemes for both model and secondary structure are the same. **d,** The model of HK97 (PDB: 3E8K)[2]. The color scheme for the domain is same as in **(c)**. **e,** The structural hallmarks of the HK97 fold can be clearly seen in VP39. The structural elements of VP39 are colored in blue while those from HK97 are colored in tan. **f,** The VP39 dimer is “fastened” by the hydrogen bonds (shown in short cyan rod). **g,** The C-terminal forms a “S”-like structure to link neighboring VP39 dimers. The interactions are primarily through hydrogen bonds and indicated by cyan dotted lines.

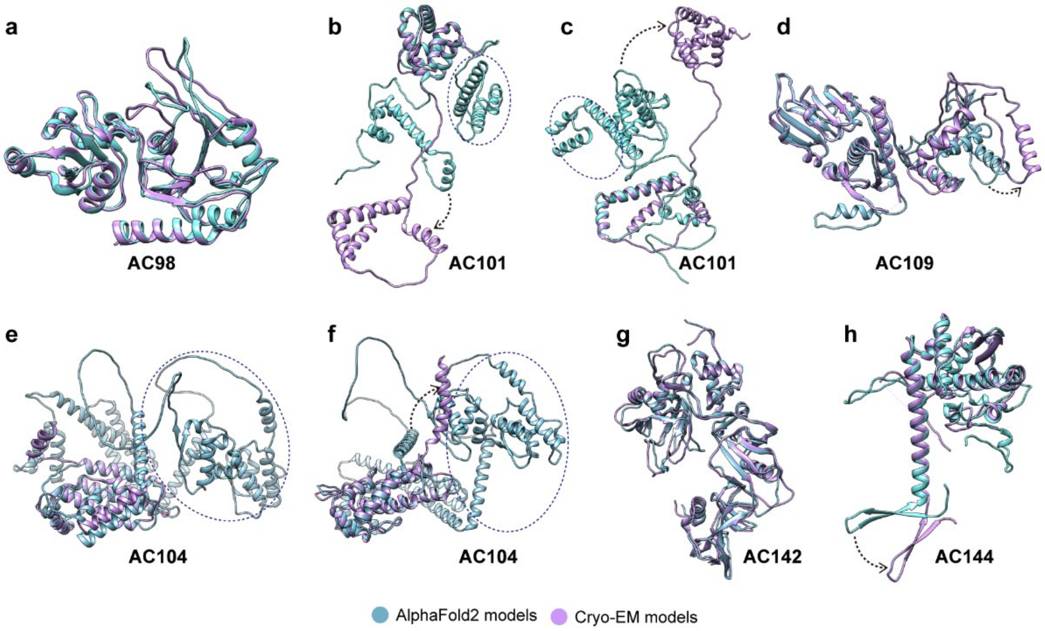

Supplementary Figure 4. Models for the base and head domains.

a, The model for AC98 from AlphaFold2 (AF2) is shown in cornflower blue while the density refined model is colored in purple. Only the loops show differences between the AF2 model before and after cryoEM density refinement. **b and c,** Model for AC101. The C-terminal domain (circle) could not be seen in the cryo-EM density map. The overall organization of the dimerization domain and the N-terminal domain (ring-like) is different between the prediction and experimental data. Models of dimerization domain (in B) and N-terminal ring-like domain were fit to the density separately. **d,** The model for AC109 from AF2 was altered at the C-terminal "C-shape" structure comparing to that from the cryo-EM density map. **e and f,** Models for AC104. The N-terminal region (circle) was not evident in the cryo-EM density map. **e,** reflects the structure for AC104-1 and AC104-2. **f,** illustrated the model for AC104-3, where the position of the long helix changes compared to that of the predicted model. **g,** The model for AC142 has little variation between the AF2 prediction and the experimental data. **h,** The model for AC144 has a small difference between the Alphafold prediction and cryo-EM data at the N-terminal "anchor".

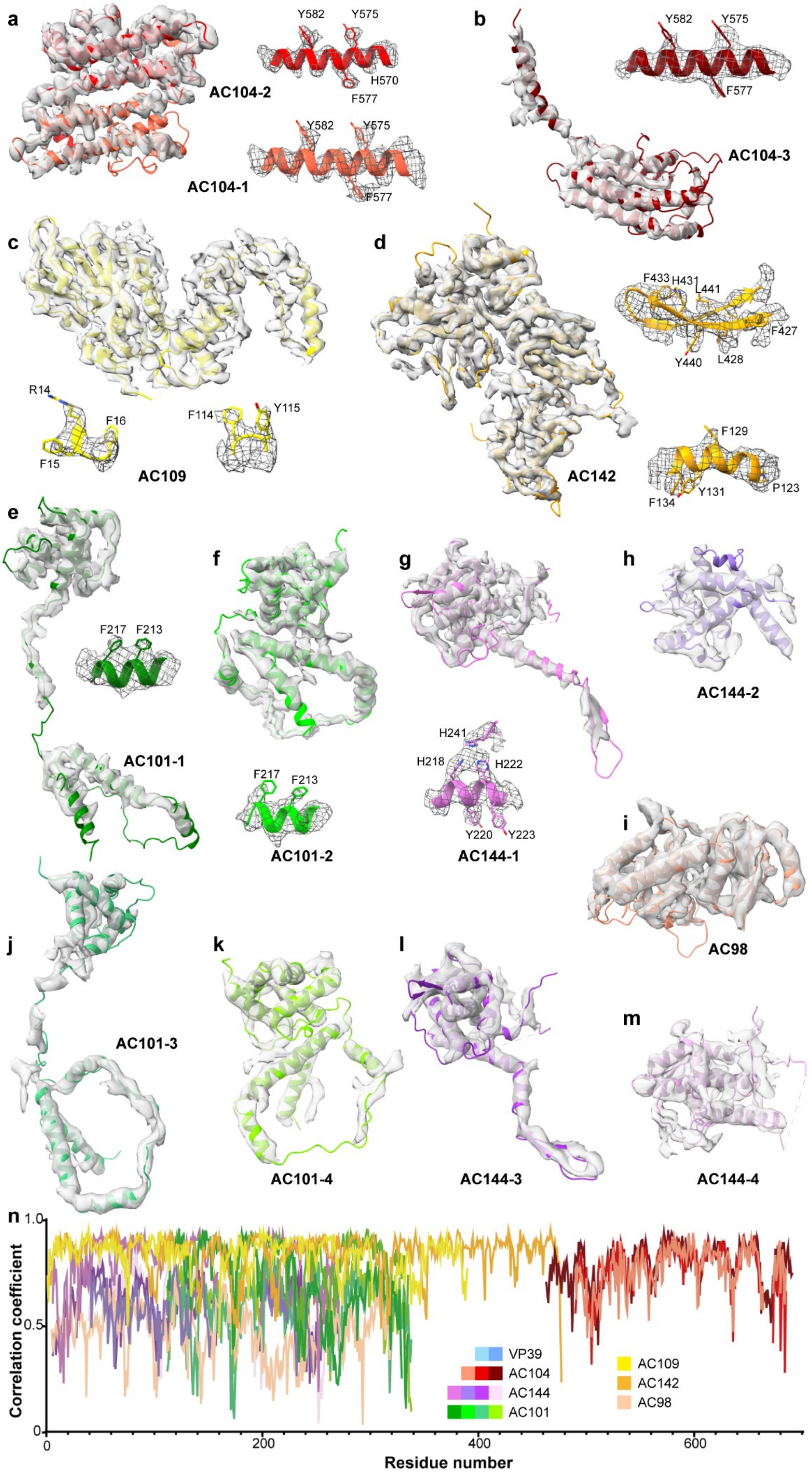

a
Y582
Y575
H570
F577
AC104-2
AC104-1
b
AC104-3
c
R14
F16
F15
F114
Y115
AC109
d
F433
H431
L441
F427
Y440
L428
F129
P123
F134
Y131
AC142
e
F217
F213
AC101-1
f
AC101-2
g
H241
H218
H222
Y220
Y223
AC144-1
h
AC144-2
i
AC98
j
AC101-3
k
AC101-4
l
AC144-3
m
AC144-4
n
Correlation coefficient
Residue number
VP39
AC104
AC144
AC101
AC109
AC142
AC98

Supplementary Figure 5. Base and head domain maps superimposed on the corresponding models.

a, Map for AC104-1 and 104-2 superimposed with model. The densities for Tyr and Phe are discernable. **b,** Map for AC104-3. Comparing with the AC104-3 map to Ac104-1 and 104-2, densities for some short loop areas are blurred. However, the densities for Tyr and Phe are identifiable. **c,** The density map of AC109 superimposed with its model. The several large sidechains are shown . **d,** The density map of AC142 superimposed with its model. The typical helix and two-stranded β-sheet are shown with the model. Again, the densities for larger sidechains for discernable. **e and f,** Maps for AC101-1 and AC101-2 together with the model are shown. The densities for long loops are missing in some areas due to flexibility. In areas rich with helices, bulky sidechains can be identified. **g,** The density map of 144-1 is shown superimposed with its model. Although the densities in some areas are discontinued, bulky sidechains can still be identified. **h, i, j, k, l, m** show the maps of AC144-2, AC98, AC101-3, AC101-4, AC144-3, and AC144-4 superimposed with their model, respectively. The resolution of these subunit maps is lower than the aforementioned protein maps but still sufficient for clearly resolving secondary structure and reliably fitting models to the density map. All density maps are shown in grey, while the models are colored as in Figure 4. **n,** shows the per-residue correlation coefficient of the refined models in the corresponding density map. The atomic models in outer shell, inner layer and plug were calculated against the corresponding local refined maps respectively.

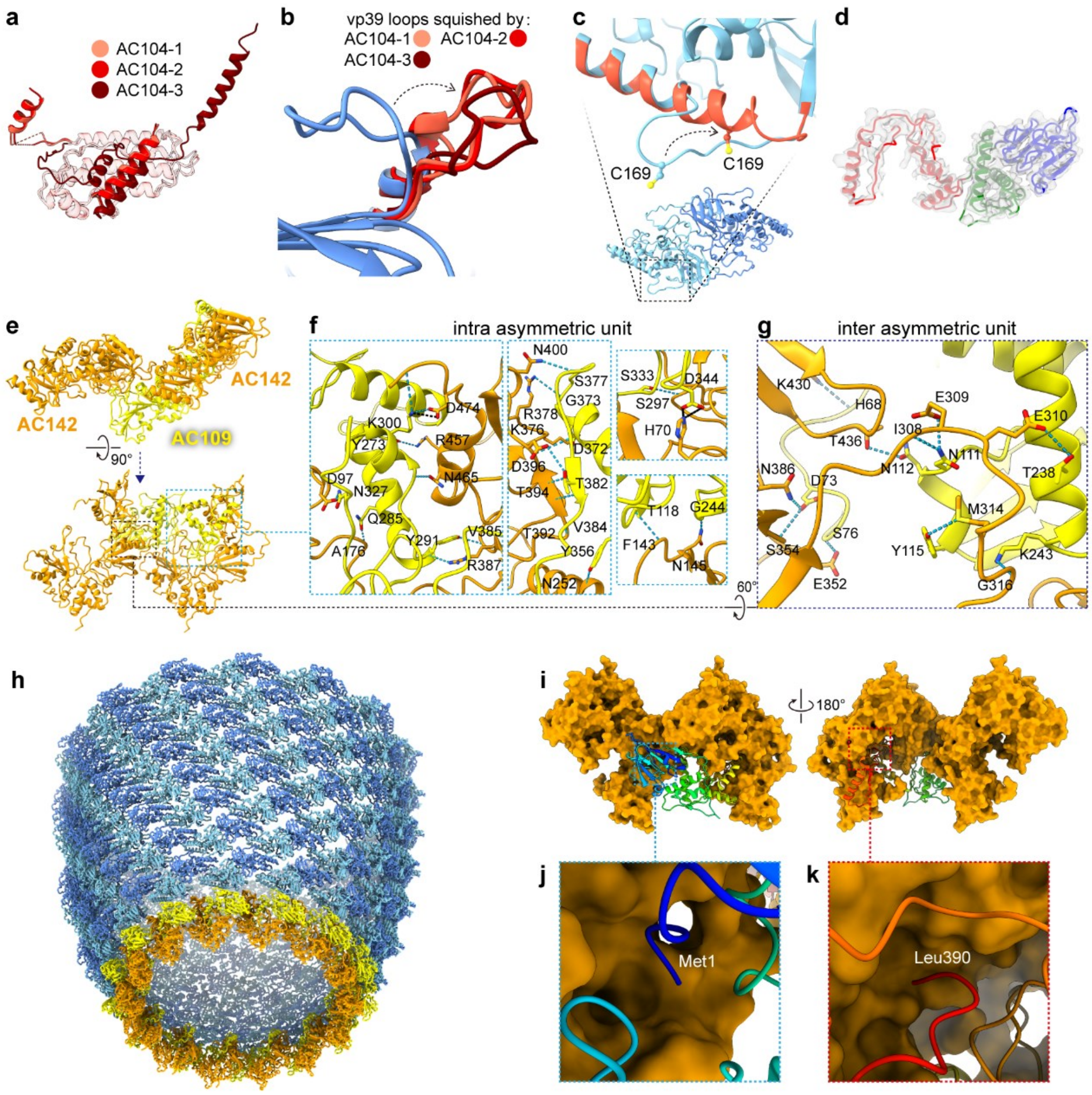

Supplementary Figure 6. Structural features of the outer shell at both ends of the AcMNPV capsid.

a, Models for AC104-1 and AC104-2 have same conformation and form a homodimer. AC104-3 has different conformation. **b,** The conformations of VP39's outmost loops are altered by AC104 at the base. Three loops (colored in salmon, red and dark red) of three VP39 at different positions (boxed in Fig. 4c with black dot lines) are flattened by AC104-1, AC104-2 and AC104-3, respectively. The outmost loop of VP39 in the helical cylinder is shown in cornflower blue for comparison. **c,** Due to the interactions with AC109, part of the loop (Thr163-Glu176) in VP39 at the end of the cylinder (colored with orchid) becomes a part of helix α_7, making the "spine helix" longer, as well as allowing C169 taking part in the formation of disulfide bond. **d,** The model of AC109 superimposed with the grey density. AC109 has a "C-shaped" C-terminal domain (colored red). The N-terminal and central region are colored with blue and green, respectively. **e,** Each AC109 binds two neighboring AC142s in both head and base. **f,** Enlarged view of the boxed area in panel e shows intra-subunit interactions between AC109 and AC142 in one asymmetric unit. The interactions primarily are through hydrogen bonds and salt bridges. **g,** Enlarged view of the corresponding area boxed in panel e shows the interface between AC109 and AC142 in neighboring asymmetric units. **h,** Fourteen AC109-AC142 hetero-dimers form a "barrel-hoop" like structure, blocking the elongation of cylinder, and tightening the VP39 cylinder (blue). AC109 is colored in yellow and AC142 is colored in orange. **i,** AC142 embraces AC109 tightly. AC142 is shown as an orange surface. AC109 is colored in rainbow color with blue at the N-terminus to red at the C-terminus. **j and k**, Enlarged views of boxed area in panel i. The first amino acid of AC109 (panel j) and the C-terminus Leu390 (panel k) are tightly packed by AC142.

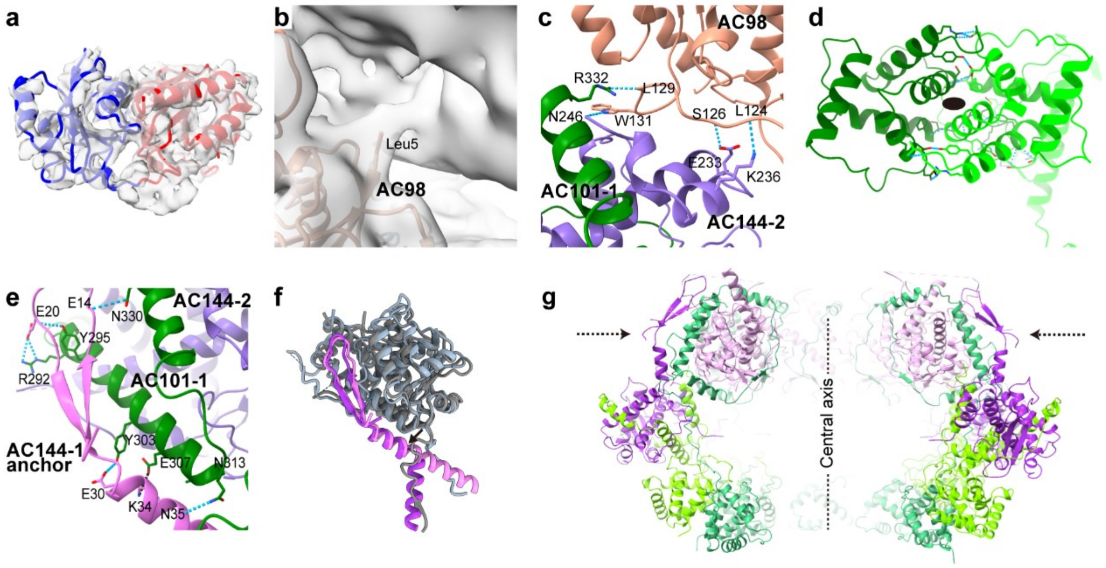

Supplementary Figure 7. Structural features of the inner layer and plug elements of the AcMNPV capsid.

a, The model for AC98 is shown superimposed with density (gray). The N-terminal domain is colored in blue and C-terminal domain, which has structural features of a haloacid dehalogenase (HAD), is colored in red. **b,** As the display threshold lowed, the interaction between the N-terminus and dsDNA strand can be found. **c,** AC98 interacts with AC144-2 and AC101-1 in the inner layer through hydrogen bonds (cyan dotted line). **d,** Two AC101 dimerization domains form a dimer through extensive interactions, including hydrogen bonds. The local 2-fold symmetry axis is indicated as a black ellipse. **e,** The N-terminal structure of AC144-1 interacts with the “ring” structure of AC101 through hydrogen bonds and a salt bridge (black dot line). AC101-1 encircles AC144-2. **f,** The N-terminus of AC144-1 (lilac), AC144-2 (gray) and “ring” structure of AC101-1 (gray) in inner layer, are aligned to AC144-3 (purple), AC144-4 (steel gray) and “ring” structure of AC101-3 (steel gray) in plug. All elements are alignment well except for the bent helix in AC144-3 (purple). **g,** The bent helix in AC144-3 (purple) in plug results in the seven AC144-4s (pink) pointing to the central axis and form a plug to seal the end of capsid. The color schemes used in panel b, c, d, e, g are same as that in Fig. 4. and Fig. 5

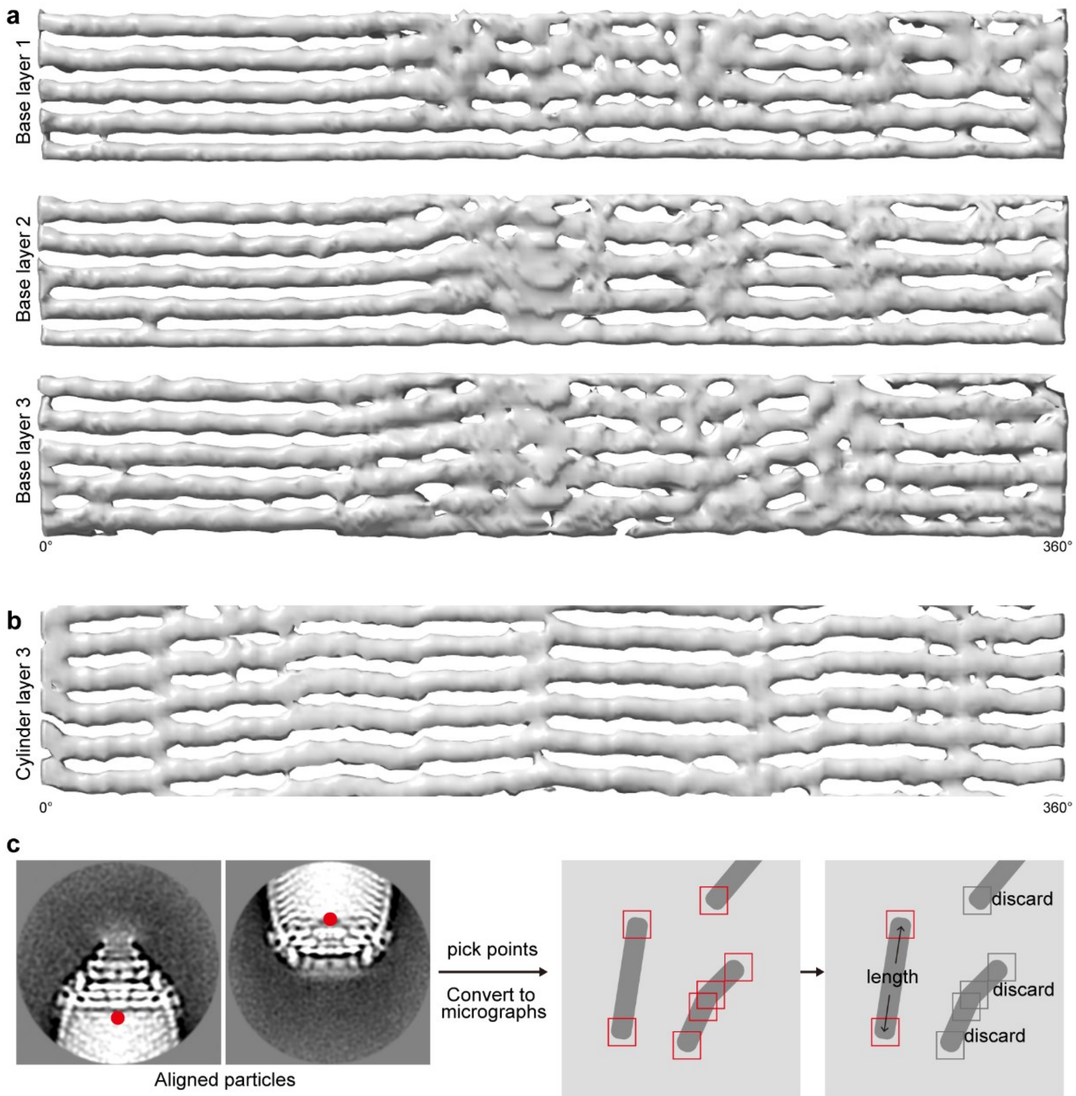

Supplementary Figure 8. The organization of genome dsDNA.

a, Planar display of unwrapped genome dsDNA strands at base shows the dsDNA strands are stacked. The outer 3 layers are shown here. **b,** Planar display of unwrapped genome dsDNA strands in the helical cylinder. The third layer of dsDNA strands is shown here. **c,** The method for calculating the length distribution of AcMNPV helical capsid is illustrated. First, the coordinates of the beginning and end of the capsid inner cavity were determined in the reconstructions. The length of the capsid inner cavity was then computed on the AcMNPV capsid in the raw micrographs. Bent and the incomplete particles were disregarded.

Supplementary Table 1. Fitting of AlphaFold2 models

Round 1											
Submap 1	Model	Ac19	**Ac144**	Ac5	Ac143	Ac85	Ac97	Ac78	Ac59	Ac137	Ac56
	CC	0.74	**0.52**	0.50	0.50	0.48	0.41	0.41	0.39	0.39	0.37
Submap 2	Model	Ac19	Ac85	Ac59	Ac151	Ac140	Ac78	Ac41	Ac121	Ac97	Ac58
	CC	0.53	0.36	0.34	0.34	0.34	0.33	0.33	0.33	0.32	0.32
Submap 3	Model	**Ac144**	Ac19	Ac111	Ac29	Ac76	Ac37	Ac137	Ac73	Ac85	Ac47
	CC	**0.61**	0.53	0.39	0.35	0.34	0.34	0.34	0.33	0.32	0.30
Submap 4	Model	Ac19	Ac85	Ac143	Ac97	Ac106	Ac5	Ac59	Ac140	Ac60	Ac29
	CC	0.58	0.53	0.44	0.43	0.43	0.40	0.36	0.36	0.35	0.35
Submap 5	Model	Ac85	**Ac98**	Ac140	Ac137	Ac78	Ac122	Ac107	Ac59	Ac43	Ac74
	CC	0.63	**0.56**	0.43	0.42	0.41	0.40	0.37	0.35	0.35	0.34
Submap 6	Model	**Ac142**	**Ac109**	Ac19	Ac78	Ac106	Ac53	Ac28	Ac52	Ac96	Ac35
	CC	**0.57**	**0.55**	0.51	0.49	0.38	0.36	0.35	0.35	0.33	0.32
Submap 7	Model	Ac19	Ac5	Ac29	Ac76	Ac85	Ac78	Ac59	Ac58	Ac53	Ac74
	CC	0.60	0.59	0.57	0.53	0.51	0.51	0.43	0.42	0.42	0.40
Round 2											
Submap 1	Model	**Ac144C**	Ac39C	Ac89C	Ac74C	Ac141aN	Ac99C	Ac88N	Ac21N	Ac148C	Ac135C
	CC	**0.39**	0.32	0.29	0.29	0.28	0.27	0.25	0.25	0.25	0.25
Submap 2	Model	Ac36N	Ac54C	Ac109C	Ac27N	Ac147C	Ac89C	Ac74N	Ac142N	Ac105N	Ac9N
	CC	0.35	0.34	0.32	0.31	0.31	0.30	0.30	0.30	0.29	0.28
Submap 3	Model	Ac39C	Ac74N	Ac9C	Ac67C	Ac10C	Ac135C	Ac40N	Ac69N	Ac21N	Ac92C
	CC	0.40	0.38	0.35	0.35	0.32	0.30	0.29	0.28	0.28	0.27
Submap 4	Model	**Ac144C**	Ac74N	Ac39C	Ac144N	Ac153C	Ac51N	Ac101N	Ac89C	Ac139N	Ac88N
	CC	**0.57**	0.34	0.33	0.32	0.31	0.30	0.29	0.28	0.28	0.27
Submap 5	Model	Ac39C	**Ac104C**	Ac74N	Ac151N	Ac92C	Ac144N	Ac69N	Ac62N	Ac135C	Ac74C
	CC	0.55	**0.48**	0.42	0.42	0.36	0.31	0.30	0.30	0.30	0.29
Round 3											
Submap 1	Model	**Ac101_101**	Ac9_176	Ac9_151	Ac141a_101	Ac101_126	Ac141a_126	Ac66_76	Ac66_51	Ac66_501	Ac9_251
	CC	**0.60**	0.59	0.59	0.55	0.47	0.47	0.46	0.46	0.46	0.44
Submap 2	Model	**Ac101_51**	Ac9_176	Ac9_151	Ac66_526	Ac147_76	Ac83_551	Ac9_126	Ac74_1	Ac74_26	Ac101_26
	CC	**0.58**	0.50	0.50	0.47	0.42	0.41	0.39	0.48	0.48	0.37
Submap 3	Model	**Ac104_526**	Ac66_76	Ac66_51	Ac9_176	Ac9_151	Ac9_126	Ac141a_101	Ac74_1	Ac104_501	Ac104_476
	CC	**0.52**	0.52	0.52	0.52	0.52	0.49	0.49	0.48	0.48	0.46
Submap 4	Model	**Ac104_526**	Ac9_101	**Ac104_501**	Ac9_151	Ac9_176	Ac91_26	Ac147_76	Ac141a_101	Ac66_551	Ac23_1
	CC	**0.58**	0.57	**0.55**	0.55	0.55	0.53	0.49	0.49	0.48	0.46
"ring"											
"ring"	Model	Ac108_16	Ac109_196	Ac144_31	Ac50_646	Ac89_121	**Ac101_241**	**Ac101_271**	Ac138_331	Ac19_31	Ac77_106
	CC	0.50	0.50	0.50	0.48	0.48	**0.47**	**0.47**	0.47	0.46	0.46

The models colored in blue are finally fitted well to the corresponding sub-maps. CC: cross-correlation coefficients between map and model.

Supplementary Table 2. Cross-linking results and potential cross-linking sites in the nucleocapsid proteins

Proteins (site*)	Peptide site
AC104(494)-AC104(482)	DIVNE**K**LQK-L**K**ALEFSK
AC104(290)-AC104(278)	LHITTIDQL**K**K-ALATL**K**NR
AC104(284)-AC104(278)	LHIT**T**IDQLKK-ALATL**K**NR
AC104(285)-AC104(278)	LHIT**T**IDQLKK-ALATL**K**NR
AC104(532)-AC104(472)	NSLETLPSAANYGSLL**K**R-**K**LEDEDFLK
VP39(163)-VP39(156)	SILDT**T**NPNTFCSR-VIHSVYATT**K**
AC144(262)-AC144(168)	TSNLLL**S**KFK-IA**K**TFGASK
**Potential cross-linking sites**	
AC104(515)-AC104(515,506,627)	
AC109(53)-AC104(497,636)	
AC142(141)-AC104(K469)	

*. Cross-linked residue.

The red bold characters with underline indicate the cross-linked amino acids in the peptides.

Supplementary Table 3. Interactions among the AcMNPV nucleocapsid proteins

		VP39	AC98	AC104	AC109	AC142	AC101	AC144
**VP39**	EM result	+ (Fig.2c-2j)	+ (Fig.5b)	+ (S.Fig.6b)	+ (Fig.4f)	-	-	-
	Reference*	-	+[3]	+[3]	-	-	-	-
	Cross-link	+	-	-		-	-	-
**AC98**	EM result		-	-	-	-	+ (Figs7c)	+ (Figs7c)
	Reference*		-	+[3]	-	-	-	-
	Cross-link		-	-	-	-	-	-
**AC104**	EM result			+ (Fig.4d)	+ (Fig.4e)	-	-	-
	Reference*			+[4]	-	-	-	-
	Cross-link			+	-	-	-	-
**AC109**	EM result				-	+ (Fig.4h, S.Fig.6e-g)	+ (Fig.4g)	-
	Reference*				-	+[5]	-	-
	Cross-link				-	-	-	-
**AC142**	EM result					+ (S.Fig.e-g)	-	-
	Reference*					-	-	-
	Cross-link					-	-	-
**AC101**	EM result						+ (Figs7d)	+ (Fig.5c,e-g)
	Reference*						-	+[6]
	Cross-link						-	-
**AC144**	EM result							+
	Reference*							-
	Cross-link							+

*, Previously reported results which support interactions between the nucleocapsid proteins.

Supplementary Table 4. Cryo-EM data collection, refinement and validation statistics

	VP39<br><br>(EMDB-35245)<br>(PDB 8I8A)	Outer shell and inner layer<br>(EMDB-35246)<br>(PDB 8I8B)	Plug<br><br>(EMDB-35247)<br>(PDB 8I8C)
**Data collection and processing**			
Magnification	105,000x	105,000x	105,000x
Voltage (kV)	300	300	300
Electron exposure (e–/Å^2)	60	60	60
Defocus range (μm)	-1 to -2.5	-1 to -2.5	-1 to -2.5
Pixel size (Å)	1.35	1.35	1.35
Symmetry imposed	C1	C1	C1
Initial particle images (no.)	2,579,934	337,988	168,994
Final particle images (no.)	1,635,004	337,988	36,127
Map resolution (Å)	3.2	4.3	4.9
FSC threshold	0.143	0.143	0.143
**Refinement**			
Initial model used (PDB code)	-	AlphaFold	AlphaFold
Model resolution (Å)	3.9	6.7	8.1
FSC threshold	0.5	0.5	0.5
Map sharpening *B* factor (Å^2)	122.9	166.4	223.9
Model composition			
Non-hydrogen atoms	4,978	30,021	7,215
Protein residues	614	3,660	879
Ligands	-	-	-
B factors (Å^2)			
Protein	89.60	124.26	122.82
Ligand	-	-	-
R.m.s. deviations			
Bond lengths (Å)	0.003	0.005	0.009
Bond angles (°)	0.605	0.935	0.947
Validation			
MolProbity score	2.19	2.10	1.99
Clashscore	11.60	12.57	8.80
Poor rotamers (%)	0.00	0.56	0.84
Ramachandran plot			
Favored (%)	87.70	92.05	91.00
Allowed (%)	12.30	7.86	9.00
Disallowed (%)	0.00	0.08	0.00

Supplementary References

1 Sun, L. *et al.* Cryo-EM structure of the bacteriophage T4 portal protein assembly at near-atomic resolution. *Nature Communications* **6** (2015). https://doi.org:10.1038/ncomms8548

2 Gertsman, I. *et al.* An unexpected twist in viral capsid maturation. *Nature* **458**, 646-650 (2009). https://doi.org:10.1038/nature07686

3 Wu, W. B. *et al.* Autographa californica Multiple Nucleopolyhedrovirus 38K Is a Novel Nucleocapsid Protein That Interacts with VP1054, VP39, VP80, and Itself. *Journal of Virology* **82**, 12356-12364 (2008). https://doi.org:10.1128/Jvi.00948-08

4 Marek, M., Merten, O. W., Francis-Devaraj, F. & Oers, M. M. Essential C-terminal region of the baculovirus minor capsid protein VP80 binds DNA. *J Virol* **86**, 1728-1738 (2012). https://doi.org:10.1128/JVI.05600-11

5 Lehiy, C. J., Wu, W., Berretta, M. F. & Passarelli, A. L. Autographa californica M nucleopolyhedrovirus open reading frame 109 affects infectious budded virus production and nucleocapsid envelopment in the nucleus of cells. *Virology* **435**, 442-452 (2013). https://doi.org:10.1016/j.virol.2012.10.015

6 Braunagel, S. C., Guidry, P. A., Rosas-Acosta, G., Engelking, L. & Summers, M. D. Identification of BV/ODV-C42, an Autographa californica nucleopolyhedrovirus orf101-encoded structural protein detected in infected-cell complexes with ODV-EC27 and p78/83. *J Virol* **75**, 12331-12338 (2001). https://doi.org:10.1128/JVI.75.24.12331-12338.2001
