# Supplementary material for: Architecture of the baculovirus nucleocapsid revealed by cryo-EM
Source: Nat Commun. 2023 Nov 18;14:7481. doi: 10.1038/s41467-023-43284-1 (PMC10657434; doi:10.1038/s41467-023-43284-1)
Supplement: Supplementary file 3 — Description of Additional Supplementary Files [file 41467_2023_43284_MOESM3_ESM.pdf]

Description of Additional Supplementary Files

File name: Supplementary Movie 1
Description: Overall structure and the VP39 helical cylinder.

File name: Supplementary Movie 2
Description: Conformational changes of VP39 at the ends of the helical cylinder.

File name: Supplementary Movie 3
Description: Overall structure of the AcMNPV nucelocapsid base.
