# Supplementary material for: Architecture of the baculovirus nucleocapsid revealed by cryo-EM
Source: Nat Commun. 2023 Nov 18;14:7481. doi: 10.1038/s41467-023-43284-1 (PMC10657434; doi:10.1038/s41467-023-43284-1)
Supplement: Supplementary file 7 — Reporting Summary [file 41467_2023_43284_MOESM7_ESM.pdf]

nature portfolio

Corresponding author(s): Xudong Jia

Last updated by author(s): Oct 16, 2023

Reporting Summary

Nature Portfolio wishes to improve the reproducibility of the work that we publish. This form provides structure for consistency and transparency in reporting. For further information on Nature Portfolio policies, see our Editorial Policies and the Editorial Policy Checklist.

Statistics

For all statistical analyses, confirm that the following items are present in the figure legend, table legend, main text, or Methods section.

n/a	Confirmed	
☐	☒	The exact sample size (*n*) for each experimental group/condition, given as a discrete number and unit of measurement
☒	☐	A statement on whether measurements were taken from distinct samples or whether the same sample was measured repeatedly
☒	☐	The statistical test(s) used AND whether they are one- or two-sided<br>*Only common tests should be described solely by name; describe more complex techniques in the Methods section.*
☒	☐	A description of all covariates tested
☒	☐	A description of any assumptions or corrections, such as tests of normality and adjustment for multiple comparisons
☒	☐	A full description of the statistical parameters including central tendency (e.g. means) or other basic estimates (e.g. regression coefficient) AND variation (e.g. standard deviation) or associated estimates of uncertainty (e.g. confidence intervals)
☒	☐	For null hypothesis testing, the test statistic (e.g. *F*, *t*, *r*) with confidence intervals, effect sizes, degrees of freedom and *P* value noted<br>*Give P values as exact values whenever suitable.*
☒	☐	For Bayesian analysis, information on the choice of priors and Markov chain Monte Carlo settings
☒	☐	For hierarchical and complex designs, identification of the appropriate level for tests and full reporting of outcomes
☒	☐	Estimates of effect sizes (e.g. Cohen's *d*, Pearson's *r*), indicating how they were calculated

Our web collection on statistics for biologists contains articles on many of the points above.

Software and code

Policy information about availability of computer code

Data collection	SerialEM 3.8
Data analysis	MotionCor 2, CTFFIND 4, SPHIRE-crYOLO 1.8.4, Relion 3.1.3, cryoSPARC 3.2, EMAN 2.91 , Chimera 1.14, ChimeraX 1.3, Coot 0.8.9.2, Phenix 1.2, Rosetta 2021.16, Alphafold 2, pLink2

For manuscripts utilizing custom algorithms or software that are central to the research but not yet described in published literature, software must be made available to editors and reviewers. We strongly encourage code deposition in a community repository (e.g. GitHub). See the Nature Portfolio guidelines for submitting code & software for further information.

Data

Policy information about availability of data

All manuscripts must include a data availability statement. This statement should provide the following information, where applicable:

- Accession codes, unique identifiers, or web links for publicly available datasets
- A description of any restrictions on data availability
- For clinical datasets or third party data, please ensure that the statement adheres to our policy

The cryo-EM maps of overall helical cylinder, apical domain and base, have been deposited in the Electron Microscopy Data Bank (EMDB) under accession codes EMD-35242 [https://www.ebi.ac.uk/emdb/EMD-35242], EMD-35243 [https://www.ebi.ac.uk/emdb/EMD-35243] and EMD-35244 [https://www.ebi.ac.uk/emdb/EMD-35244], respectively. The atomic coordinates were deposited in the RCSB Protein Data Bank (PDB) under the accession codes 8I8A [http://doi.org/10.2210/

pdb8I8A/pdb] (VP39), 8I8B [http://doi.org/10.2210/pdb8I8B/pdb] (outer shell and inner layer) and 8I8C [http://doi.org/10.2210/pdb8I8C/pdb] (plug), with their maps in EMDB under accession code EMD-35245 [https://www.ebi.ac.uk/emdb/EMD-35245], EMD-35246 [https://www.ebi.ac.uk/emdb/EMD-35246] and EMD-35247 [https://www.ebi.ac.uk/emdb/EMD-35247], respectively. Previous reported atomic models of T4 phage and HK97 can be accessed under PDBID accession codes 3JA7 [http://doi.org/10.2210/pdb3JA7/pdb] and 3E8K [http://doi.org/10.2210/pdb3E8K/pdb]. The length distribution raw data presented in Supplementary Fig. 2d in this study is provided in the Source Data file. Source data are provided with this paper. The data that support this study are also available from the corresponding authors upon reasonable request.

Research involving human participants, their data, or biological material

Policy information about studies with human participants or human data. See also policy information about sex, gender (identity/presentation), and sexual orientation and race, ethnicity and racism.

Reporting on sex and gender	Not applicable.
Reporting on race, ethnicity, or other socially relevant groupings	Not applicable.
Population characteristics	Not applicable.
Recruitment	Not applicable.
Ethics oversight	Not applicable.

Note that full information on the approval of the study protocol must also be provided in the manuscript.

Field-specific reporting

Please select the one below that is the best fit for your research. If you are not sure, read the appropriate sections before making your selection.

☒ Life sciences ☐ Behavioural & social sciences ☐ Ecological, evolutionary & environmental sciences

For a reference copy of the document with all sections, see nature.com/documents/nr-reporting-summary-flat.pdf

Life sciences study design

All studies must disclose on these points even when the disclosure is negative.

Sample size	The sample sizes (2,006 raw micrographs) was chosen, considering the highly symmetry of our helical sample, to reach 3-4 Å resolution reconstructions.
Data exclusions	Cryo-EM micrograph with heavy contamination and motion were excluded. Particles were excluded based on their general appearance and the number of particles in each set. This is genera practice in our field. (e.g. Scheres S.H.W, RELION: Implementation of a Bayesian approach to cryo-EM structure determination. Journal of tructural Biology 2012 and Scheres S.H.W. Chapter Six - Processing of Structurally Heterogeneous Cryo-EM Data in RELION. Methods in Enzymology 2016).
Replication	As single particle analysis is an averaging method, it is not usual to perform replicates, and hence no replication was performed. Sample was screened many times at mid-end cryo-EM before final data collection, showing similar results.
Randomization	Data processing were start with all particles in random sets and oritation. Software (Relion and cryoSPARC) uses a reference free algorithm for 2D classification. Randomization, other than this, is not generally used in this type of study.
Blinding	Blinding of the single particle cryo-EM data would not have been technical feasible.

Reporting for specific materials, systems and methods

We require information from authors about some types of materials, experimental systems and methods used in many studies. Here, indicate whether each material, system or method listed is relevant to your study. If you are not sure if a list item applies to your research, read the appropriate section before selecting a response.

Materials & experimental systems

n/a	Involved in the study	
☒	☐	Antibodies
☐	☒	Eukaryotic cell lines
☒	☐	Palaeontology and archaeology
☐	☒	Animals and other organisms
☒	☐	Clinical data
☒	☐	Dual use research of concern
☒	☐	Plants

Methods

n/a	Involved in the study	
☒	☐	ChIP-seq
☒	☐	Flow cytometry
☒	☐	MRI-based neuroimaging

Eukaryotic cell lines

Policy information about cell lines and Sex and Gender in Research

Cell line source(s)	ATCC(Sf9), ThermoFisher(Sf9 Cells in Grace's, B82501)
Authentication	None of the cell lines used were authenticated
Mycoplasma contamination	Cell lines were not tested for mycoplasma contamination
Commonly misidentified lines (See ICLAC register)	No

Animals and other research organisms

Policy information about studies involving animals; ARRIVE guidelines recommended for reporting animal research, and Sex and Gender in Research

Laboratory animals	Spodoptera exigua, third instar larva
Wild animals	The study did not involve wild animals
Reporting on sex	Sex was not considered in study design. Virions produced by both sex of spodoptera exigua larva were mixed.
Field-collected samples	The study did not involve samples collected from the field.
Ethics oversight	No experiments were performed on live vertebrates and/or higher invertebrates in our study. No ethical approval or guidance was required.

Note that full information on the approval of the study protocol must also be provided in the manuscript.

Plants

Seed stocks	Not applicable.
Novel plant genotypes	Not applicable.
Authentication	Not applicable.
